# The Advancement of Neutron-Shielding Materials for the Transportation and Storage of Spent Nuclear Fuel

**DOI:** 10.3390/ma15093255

**Published:** 2022-04-30

**Authors:** Zhengdong Qi, Zhong Yang, Jianping Li, Yongchun Guo, Guichun Yang, Yang Yu, Jiachen Zhang

**Affiliations:** 1 School of Materials and Chemical Engineering, Xi’an Technological University, Xi’an 710021, China; qizhengdong@st.xatu.edu.cn (Z.Q.); jpli-0416@163.com (J.L.); guoyc@xa-tu.edu.cn (Y.G.); zjc18329374978@163.com (J.Z.); 2 School of Electrical and Mechanical Engineering, Xinjiang Institute of Technology, Aksu 843100, China; 3 State Key Laboratory of Engines, Tianjin University, Tianjin 300192, China; ygc2001@163.com; 4 Xi’an Sunward Aeromat Co., Ltd., Xi’an 710021, China; yuyang@aeromat.cn

**Keywords:** spent nuclear fuel, transportation and storage, shielding materials, neutron absorption

## Abstract

In this paper, the mechanism of neutron absorption and common reinforced particles is introduced, and recent research progress on different types of neutron-shielding materials (borated stainless steels, B/Al Alloy, B_4_C/Al composites, polymer-based composites, and shielding concrete) for transportation and wet or dry storage of spent fuel is elaborated, and critical performance is summarized and compared. In particular, the most widely studied and used borated stainless steel and B_4_C/Al composite neutron-absorption materials in the field of spent fuel are discussed at length. The problems and solutions in the preparation and application of different types of neutron-shielding materials for spent fuel transportation and storage are discussed, and their research priorities and development trends are proposed.

## 1. Introduction

With the neutrality of the energy problems and requirements of the new policy of carbon peaking and carbon neutralizing, China’s nuclear power industry has developed rapidly. As of April 2020, there were 442 nuclear power reactors in operation for 30 countries and 220 research reactors in 53 countries, producing an average of 11,300 tons of spent fuel per year. By 2021, the installed capacity of nuclear power units under construction in China has ranked first in the world. It is estimated that the annual spent fuel produced by China’s nuclear power plants is expected to exceed 2820 tons in 2030, but China’s current nuclear power plants are designed to store spent fuel for 10 years, so the safe disposal of spent fuel is a great challenge for China. In addition, with a large number of early nuclear power reactors shutting down one after another, the global nuclear industry is expected to usher in the best part of the first round of decommissioning in the next 15 to 20 years. The safe transportation and storage of a large amount of spent fuel is an unavoidable issue. Although nuclear power is a safe, clean, and economical new energy source, the spent fuel produced by nuclear reactors will fission to produce strong neutron and gamma-ray radiation spontaneously, which causes serious harm to people and the surrounding environment. It must be disposed of properly. Different from the fast-growing nuclear power industry, the spent fuel processing industry has developed slowly, resulting in the accumulation of a large amount of spent fuel in operating nuclear power plants.

**Figure 1:**
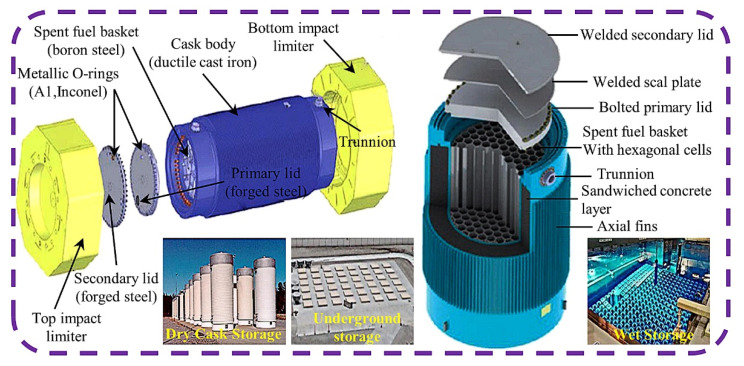
Typical spent fuel transport barrel and storage [2].

There are two main ways to deal with spent fuel in the world. One is the “one cycle through” strategy, which belongs to an open cycle, that is, the spent fuel is directly cooled, solidified, encapsulated, and deeply buried. The representative countries adopting this method include the United States, Canada, Australia, etc. Second, the “post-treatment” strategy pertains to a closed cycle, which is first stored in a pool for a certain period of time until the radioactivity and waste heat are reduced to a certain extent, then transported to storage facilities for storage, or directly to the reprocessing plant to recover valuable substances. The representative countries are China, France, The United Kingdom, Russia, and so on. This method requires not only a large number of spent fuel storage racks, but also a large number of spent fuel transportation containers in the process of spent fuel transportation, off-reactor storage, and reprocessing. In order to shield radioactivity, the spent fuel container has an outer shell up to 12~38 cm thick, which is composed of multiple layers of the following materials: steel, concrete, lead, boronized polymer, etc. (Figure 1). Under these materials and this thickness, the full load of a spent fuel container used for road transportation is as high as 25 tons, of which the spent fuel is only 0.5~2.2 tons, and the container used for railway transportation has a full-load weight of 150 tons and can carry 20 tons of spent fuel [1]. Neutron-absorbing materials for spent fuel transportation and storage are the core materials used to make spent fuel storage grids and containers. The transportation and storage of spent fuel plays an important role in the safe disposal of spent fuel, and neutron-absorption materials are the core materials for the storage and application of spent fuel. Therefore, the development of advanced neutron-absorption materials is helpful to increase the transportation and storage capacity of spent fuel, reduce the cost, and improve the disposal capacity of spent fuel.

At present, there are five main types of neutron-absorption materials for spent fuel storage and transportation, which are used for critical control in spent nuclear fuel storage and transportation, namely, boron stainless steel, boron aluminum alloy, aluminum-based boron carbide, polymer-based shielding materials, and shielding concrete. These materials have the following three functions in spent fuel storage and transportation: (1) structural support, (2) geometric control of spent fuel assemblies, and (3) nuclear radiation criticality safety. Boron stainless steel and aluminum-based boron carbide are available for wet and dry storage applications. Boron stainless steel and aluminum-based boron carbide can be used in dry–wet storage applications. Boron stainless steel is usually the preferred material for the manufacture of spent fuel storage framework and storage basket. It mainly acts as a structural material to keep the subcritical geometric arrangement of the stored spent fuel assembly [3]. Aluminum-based boron carbide is usually used as a neutron absorber to maintain the subcriticality of the fuel assembly by absorbing thermal neutrons [4]. Boron–aluminum alloy is only used for dry storage system canister designs. Polymer composites have often been used as neutron moderators to slow down the neutron motions due to their high volume fraction of hydrogen atoms and are often used in wet storage [5]. Shielded concrete is mainly used for radiation-proof building materials or shielding fillers in transport containers in dry storage [6]. It can significantly reduce the penetration strength of neutron flow in spent fuel storage and keep the structure stable.

## 2. Neutron-Absorption Elements and Shielding Mechanism

Among the neutron-absorption elements, gadolinium (Gd), cadmium (Cd), samarium (Sm), boron (B), europium (Eu), and dysprosium (Dy) have higher macroscopic neutron-absorption cross sections [7]. The maximum neutron-absorption cross sections of ^155^Gd and ^157^Gd are 49,700 barn, and abundances in natural Gd are 14.8% and 15.7%, respectively. The poor mechanical properties and the preparation environments are stringent due to virulence and carcinogenicity [8]. The neutron-absorption cross sections of ^149^Sm and ^152^Sm are 5922 barn, and the abundances are 13.8% and 26.7%, which are slightly toxic. The fine-powder state of Sm can cause spontaneous combustion, and Sm is easy to magnetize and difficult to demagnetize. The neutron-absorption cross section of ^151^Eu is 4530 barn, abundance is 47.8%, and the chemical properties are active and subject to much instability. The neutron-absorption cross section value of ^113^Cd is 2520 barn, and the abundance in natural Cd is 12.3%. It is highly toxic and flammable with low melting point (only 320.9 °C). ^10^B has a better thermal neutron-absorption cross section of 767 barn, and the abundance is 19.9% [9]. Table 1 is cross-sections values of a common neutron absorber in the spent fuel field. In the case of neutrons, for each element a cross section is distinguished for a given type of reaction, i.e., scattering cross section σ_s_, including coherent σ_cs_ and incoherent σ_is_ and absorption cross section σ_a_ [7].

Fillers for neutron shielding are based on the use of compounds that incorporate elements with a high thermal neutron capture cross section, which are typically added to polymeric, metal, alloy, or concrete matrices, in order to obtain more effective shielding and cost economy. However, the use of fillers may also lead to the improvement or loss of physical and mechanical properties. At present, there are two commonly used fillers: boron compounds and rare earths. The properties of several main neutron-absorbing filler materials are shown in Table 2.

Neutrons are classified into thermal neutrons (0.0253 eV), slow neutrons (0.0253 eV~1 KeV), medium-energy neutrons (1 KeV~100 KeV), fast neutrons (100 KeV~10 MeV), and high-energy neutrons (>10 MeV) according to their energy magnitude [19]. There are three modes of interaction between incident neutrons and atomic nuclei of matter elements, namely, elastic scattering, inelastic scattering, and radiation trapping. High-energy neutrons are moderated into thermal neutrons through elastic scattering from light nuclei and inelastic scattering from heavy nuclei, and the moderated thermal neutrons are absorbed by the element target nuclei with a high thermal neutron-absorption cross section. Secondary gamma rays are generated during the interaction between neutrons and target nuclei, which are usually shielded by elements with higher atomic numbers.

## 3. Neutron-Shielding Materials

### 3.1. Borated Stainless Steels

Boron steel is an alloy steel with Fe as the main base element; when the B content is more than 0.1 wt% boron steel it is called high-boron steel. Borated stainless steel is a special boron steel formed by added B to the stainless steel base [20]. Compared with other shielding materials, boron steel has stable structure at high temperature, good corrosion resistance, and good shielding effect on gamma rays [21], which makes it have comprehensive shielding properties of neutrons and gamma rays at the same time. Recently, Fe-based alloys including steels have been widely considered as realistic candidates for structural materials in spent nuclear fuel applications, such as NeutroSorbPLUS, NAS8R10, NAR-304BN, and other grades of boron steel that have been widely used in spent fuel pool shelves, storage baskets, and transport containers.

**Figure 2:**
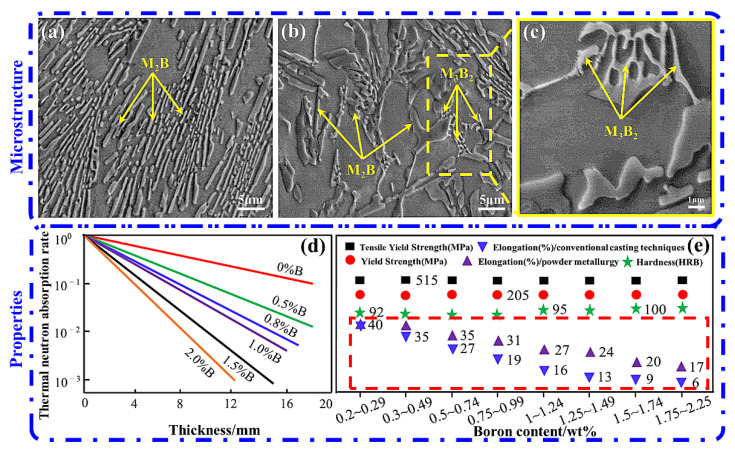
(**a**) Boride in 304 stainless steel, (**b**) boride in 316 stainless steel, and (**c**) M_3_B_2_ of high magnification [23]; (**d**) variation of thermal neutron absorptivity with B content in borated stainless steels [22]; (**e**) variation of elongation with boron content in borated stainless steels [24].

The solubility of B in α-Fe is only 0.0021%, and the solubility in γ-Fe is only 0.0082% at room temperature [22]. As shown in Figure 2a–c, when a high content of B is added to the steel for alloying, high hardness and low melting point will be formed at the grain boundary. The reticulated boride M_2_B (M is Fe, Cr, Mn) or M_3_B_2_ (M is Fe, Mo) with a length of about 20 um can easily cause serious edge cracks and sharp decrease in plasticity and toughness of boron steel during processing [23]. As shown in Figure 2d,e, the B content in boron steel is directly proportional to the thermal neutron absorptivity, and inversely proportional to the elongation [22,24]. When the B content reaches 2.25 wt%, the elongation of boron steel is only 6%, while boron steel with boron content higher than 2.25 wt% is almost too brittle to be used.

In order to improve the machinability and plasticity of boron steel, the high-boron steel used in spent fuel storage is mainly manufactured by rapid solidification technology and powder metallurgy. Compared with the boron steel produced by ordinary casting, the boron content of these processes is slightly higher, the boride distribution is more uniform, and the mechanical properties and corrosion properties are better, but the solidification behavior of boride in boron steel is not very clear. Powder metallurgy has the disadvantages of long production cycle, low efficiency, and high cost. How to improve or eliminate the reticulated borides, make the boride layer evenly distributed, and improve the plastic toughness of high-boron steel is the focus of research [19].

**Figure 3:**
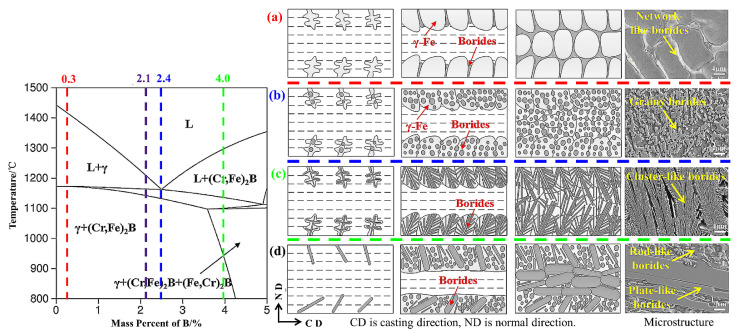
Pseudo-binary phase diagram of Fe–B and diagrams of solidification process of 0.3 wt% B (**a**), 2.4 wt% B (**b**), 4.0 wt% B (**c**) and solidification process of central microstructure (**d**) [25].

Boron content has a great influence on the solidification behavior and microstructure of steel; shown in Figure 3 is the solidification behavior of B in austenitic steel. Shown in Figure 3a, in the hypoeutectic steel with 0.3 wt% B content, the pro-eutectic γ-Fe particles are nucleated with a large number of Ni and Mn solid solution atoms in the molten steel, and they gradually grow. At the same time, a large number of B and Cr atoms are continuously squeezed into the residual liquid steel. When the B content in the residual liquid steel reaches eutectic composition, a large number of chromium-rich borides are produced at the γ-Fe grain boundary. As the solidification continues, the grain growth of γ-Fe is completed, and the boride on the grain boundary is forced to form a network of borides. Although the B content is higher in the 2.1 wt% hypoeutectic steel, due to the fast cooling rate, the boron atoms do not have enough time to fully diffuse in the molten steel, which limits the coarsening of borides and the segregation at grain boundaries. Shown in Figure 3b, in the eutectic steel with 2.4 wt% B content, the dendrites consisting of γ-Fe and borides nucleated at the surface of molten steel, and gradually grew into a fully micro-eutectic microstructure along the direction of the temperature gradient. At the last stage of solidification, the eutectic structures originating from both sides contacted each other at the central region. Fine cluster-like borides and γ-Fe solidified as intergrowth lamellar structure. Shown in Figure 3c, in the hypereutectic steel with B content of 4.0 wt%, the pro-eutectic phase is flaky boride instead of γ-Fe particles, Cr and B atoms are depleted first, and Fe, Ni, and Mn atoms are expelled to the remaining steel. In the molten steel, when the boron content in the remaining molten steel drops to the eutectic composition, rod-shaped borides containing a large amount of Fe, Ni, and Mn atoms are formed. In addition, shown in Figure 3d, the coagulation process of the central tissue cannot be ignored. This is because a large number of alloy atoms are always discharged into the residual molten steel in the central region during the solidification process of the outer layer structure. The cooling rate of the central region is relatively slower than that of the outer regions, and severe coarsening of sheet-like borides is observed in the central tissue, which is forced to grow horizontally because the outer tissue on both sides has been completely solidified [25].

**Figure 4:**
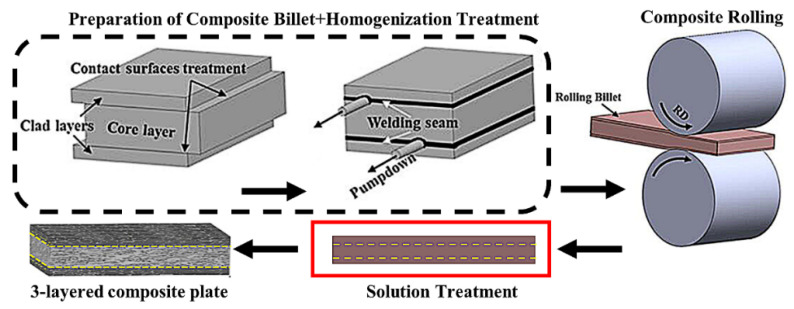
The schematic diagram showing the preparation of B steel composite plate [26].

Boron steel has high strength but low elongation at break. Composite rolling is becoming a new processing technology with excellent combination properties, such as malleability, processability, corrosion resistance, and so on. In this technology, alternating layers of soft and hard materials are bonded together to obtain laminated composite plates (Figure 4), which can load more neutron-absorption fillers and obtain more ideal elongation and strength than single-layer plates. The study shows that the elongation of the three-layer composite plate prepared by composite rolling of 1.6 wt% B stainless steel and two-layer boron-free steel is 2.5~3.0 times higher than that of the non-composite plate. The main reason for the increase in tensile elongation is that the potential necking during tensile deformation will be geometrically limited by the bonding interface. The tensile force in the potential necking area of the plate core is suppressed by the additional compressive stress applied by the ductile boron-free cladding layer, and additional tensile stress will be generated inside the cladding layer to compensate for the insufficient elongation of the plate core. Therefore, the high-boron-steel layer in the composite plate may be further deformed with the increase of tensile load [26].

**Figure 5:**
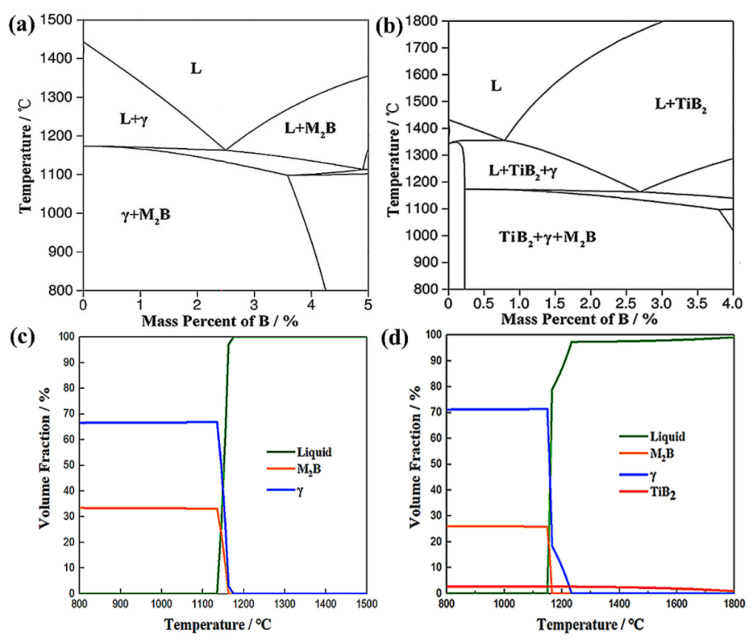
Pseudo-binary Fe–B phase diagrams based on the Ti-adding (**a**) and Ti-free steels (**b**) in the temperature range of 800~1500 °C, volume fraction diagrams of stable phases for the Ti-adding (**c**) and Ti-free steels (**d**) [33].

The plasticity of the boron steel can be improved by adding Ti (or Zr, V). Figure 5 shows the Fe–B pseudo-binary phase diagram and the volume fraction of the stable phase (M_2_B) in the boron stainless steels Ti-adding and Ti-free. The solidification paths of two steels were considerably different. From the diagram, it can be seen that the priority reaction between Ti and B reduces the combination of B and Fe, and TiB_2_ precipitates, before forming γ-Fe and M_2_B boride. These TiB_2_ particles consume a large number of boron atoms in advance, resulting in the eutectic point of the steel moving to the right. As a result, the volume fraction of M_2_B boride decreases obviously, the amount of reticulated boride precipitated at the grain boundary is reduced, and the part of boride in the network state is discontinuous, so the elongation and plasticity and toughness of the alloy are improved [27,28,29]. Different types of boron phases can be obtained by adjusting the Ti/B ratio, and the type of boron phase in the steel change process is as follows: (Fe,Cr)_2_B→(Fe,Cr)_2_B + TiB_2_→TiB_2_. When TiB_2_ completely replaces (Fe,Cr)_2_B and excessive Ti is added, the improvement of effect on borides is not obvious [30,31]. It has also been reported that the optimal mass fraction of Ti is about (2.2B + 1~1.5)% [32]. When the atomic ratio of Ti to B is 0.5, almost all of the B is transformed into TiB_2_ and uniformly distributed in the Fe matrix, and the tensile elongation and impact toughness increase [30].

**Figure 6:**
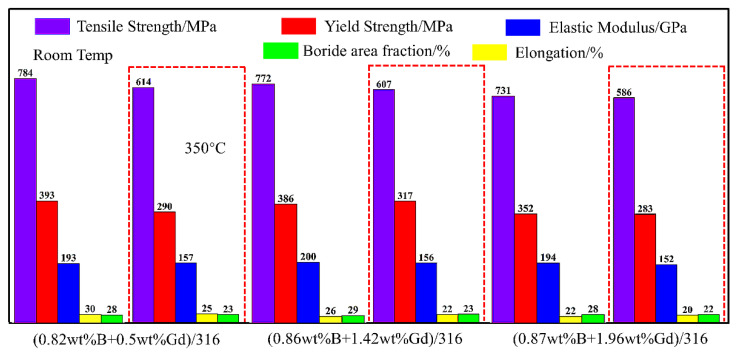
Mechanical properties of 316 stainless steel alloy containing Gd and B [35].

The addition of rare earth can also improve the plasticity of boron steel, and the addition of rare-earth element Gd with the largest neutron-absorption cross section can significantly improve the neutron-absorption properties while reducing the boride content. Gadolinium compounds do not consume chromium in the matrix, which helps to improve the corrosion resistance. At the same time, the increase of gadolinium compounds reduces the total area fraction of boride, which improves the bending properties of materials. Research shows that the addition of Gd particles to 316 L austenitic stainless steel can effectively improve the neutron-absorption properties, Gd particles can be uniformly distributed in the 316 L matrix, and the interface is well bonded. The tensile strength of 1 wt% Gd/316 L alloy steel can reached 388 MPa, the elongation reached 11%, and the thermal neutron-shielding rate was more than 95% [34]. When 0.02 wt% Gd was added to the duplex stainless steel, the neutron absorptivity is equivalent to that of the commercial 1.2 wt% boron stainless steel, the tensile strength reached 700.2 MPa, the elongation reaches 38.1%, and it has good plasticity and toughness. When Gd and B were added to stainless steel at the same time, in order to ensure the excellent mechanical properties of alloy steel, it is appropriate for Gd content to be higher than B content and B content should not exceed 1 wt%. As shown in Figure 6, when the B content in 316 stainless steel matrix is less than 1 wt%, with the increase of Gd content, the mechanical properties of alloy steel at room temperature and 350 °C (the maximum temperature limit of spent fuel storage is 350 °C) [35] meet the current standard specification of nuclear borated stainless steels.

**Figure 7:**
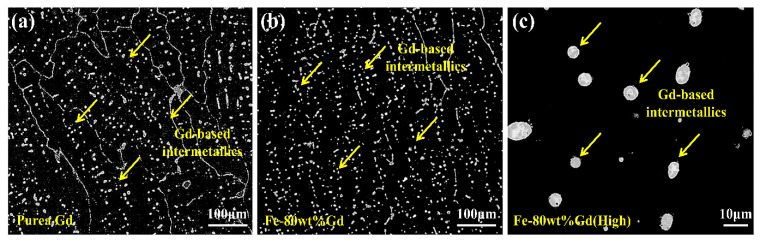
BSE images of 1 wt% Gd/2205DSS stainless steel alloy by (**a**) pure Gd, (**b**) Fe–80 wt% Gd, and (**c**) the high magnification of Gd-based intermetallic compounds [37].

Although the addition of Gd can effectively improve the neutron-absorption properties of boron steel, the solubility of Gd in stainless steel is also very small. Excessive addition of Gd can easily lead to the agglomeration of Gd at the grain boundary or the formation of larger Gd compounds in the matrix, which will lead to a sharp decline in the mechanical properties of alloy steel. For example, when Gd is added to 316 stainless steel, Gd exists mainly in the (Fe,Ni,Cr)_3_Gd intermetallic compound formed by peritectic reaction, and the melting point of the Gd-based intermetallics is about 1060 °C, so the hot workability of this kind of alloy is limited, such as hot rolling and hot forging. In addition, the wide solidification temperature range (360~400 °C) of the Gd-based intermetallics makes this kind of alloy have poor crack resistance and poor weldability, so that it cannot be used in spent fuel transportation and storage [36]. Fe–Gd master alloy is considered to be an effective way to solve this problem, especially when Fe–80 wt% Gd is added as a master alloy to unembroidered steel; the average size and distance between Gd-based intermetallics in the matrix are obviously reduced, the distribution is more uniform, and the morphology of Gd-based intermetallics is more spherical than that of pure Gd (Figure 7), Fe–Gd master alloy shows a good application prospect in the field of spent fuel [37].

Some studies have pointed out that the hot working range of borated stainless steels is narrow, which is greatly affected by hot working. When the B content is more than 1 wt%, edge cracks will appear in rolling below 1000 °C. When rolled at 1150 °C and above, a large number of borides are precipitated at the grain boundary, causing the material properties to deteriorate sharply, and the ductility of the alloy greater than 1200 °C is almost zero [38]. Based on the above analysis, it is concluded that the hot rolling temperature should be kept at 1050 °C. When the rolling deformation is more than 60%, the hot-rolled sheet has been basically transformed into as-processed structure, the material is dense, and the boride is fine and uniformly distributed [39].

Boride materials produce helium bubbles after absorbing neutrons, which affect material properties and cause further degradation of the material during long-term storage [40]. It is a fatal disadvantage to boron stainless steel, and therefore, boron stainless steel is usually used as a structural material for spent fuel storage by splicing process rather than welding process.

### 3.2. B/Al Alloy

B/Al alloy is formed by adding element B to the matrix aluminum alloy by melting or powder metallurgy. B/Al alloy has a low density, light weight, high thermal conductivity, good corrosion resistance, and almost no internal shrinkage defects. When B is added to the aluminum matrix, it is easy to form boron-rich boride at the grain boundary, which reduces the toughness of the alloy, while too little B content limits the neutron-absorption properties of the alloy. It is known from research that the high B/Al alloy containing 10 wt% to 50 wt% boron has good neutron-absorption properties, but a large number of borides are formed in the matrix, resulting in extremely poor mechanical properties of the alloy, which can only be used as functional materials [41]. In order to ensure that B/Al alloy has high B content and does not cause serious deterioration of material machinability due to the addition of excessive B, enrichment ^10^B is usually added to the alloy, for example, the Eagle-Picher company has added boron-enriched ^10^B (>95 wt%) to AA1100, AA6351 aluminum alloys to develop two types of B/Al alloy for neutron absorption in spent fuel storage buckets (Table 3). AA1100 is relatively soft and not used in structural applications and AA6351 is a structure functional material, which has good corrosion resistance in deionized water at 80 °C, but the enrichment of ^10^B alloy is complicated and expensive [42]. Ti has better affinity to B and acts as a boride-rich refinement agent to make B/Al alloy microstructure more isotropic. Layered Ti–Al metal composites show higher plastic toughness than the two components [43]. The design of this layered structure provides a way to improve the plastic toughness of B/Al alloy.

### 3.3. B_4_C/Al Composite

B_4_C/Al matrix composite is made of pure or alloy aluminum as matrix and adding B_4_C particles. Pure or alloy aluminum is widely used as matrix material because of its light weight, high strength, good toughness, and corrosion resistance. AA6061 in aluminum alloy especially has the advantages of high content of Si and Mg, good casting fluidity and low price [44]. At present, AMMC, Boralcan, Metamic, Structural Poison Tube, and other B_4_C/Al-based neutron-absorption materials have been approved to be widely used in various fields of spent fuel transportation and storage.

**Figure 8:**
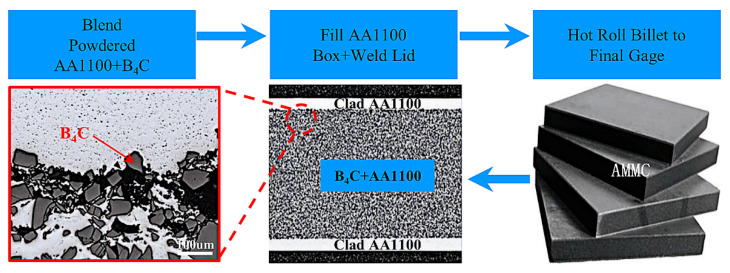
AMMC manufacturing processes [45].

AMMC is a composite non-structural neutron-absorption material produced by 3M based on boral alloy. In 2018, AMMC was qualified for use for the first time and was used in spent fuel pools for wet storage. AMMC manufacturing processes and structures are shown in Figure 8. The mixed powder containing aluminum powder and B_4_C powder is coated in an aluminum plate box, and then hot rolled to obtain a layered composite material. In this process, the highest content of B_4_C can reach 45 wt%, and the aluminum plate can be AA1000, AA5000 series aluminum. The composite plate has low porosity, good bonding between coating and center layer, strong corrosion resistance, and high thermal conductivity. The sandwich structure does not dissolve B_4_C in aluminum matrix so as to overcome the problem of limited solubility of boron in aluminum and achieve higher boron load. As a result, better neutron-absorption properties can be obtained.

**Figure 9:**
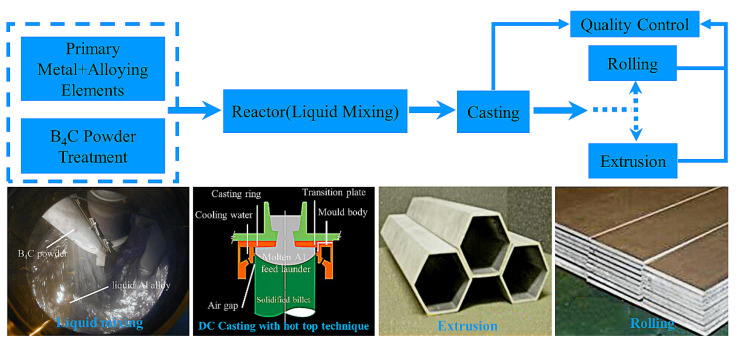
Boralcan production process route [46].

Boralcan is a neutron-absorbing material produced by Rio Tinto Alcan Company. Figure 9 shows the Boralcan manufacturing process. B_4_C powder is added together with a small amount of titanium powder in continuously stirred molten aluminum liquid, after which the molten mixture is cast into billets, and then the desired product specifications are obtained by extrusion and rolling. The base aluminum alloy in the material can be AA3004, AA6061, AA6351, and AA1100, and the content of B_4_C powder is in the range of 4.5~28.5 vol%. The material has the characteristics of uniform distribution, high hardness and toughness, weldable by friction stir welding, and strong corrosion resistance. It has been used in spent fuel dry and wet storage baskets for more than 10 years.

B_4_C/Al-based neutron-absorbing materials have a relatively good industrial foundation in China, such as B_4_C/Al-based neutron-absorbing materials produced by Antai Nuclear Sinogen New Material Technology Co., Ltd. (Beijing, China). B_4_C content is up to 10~35 wt%, and the specifications include plates, bars, tubes, and various kinds of hetero-types. The content of B_4_C in neutron-absorption materials developed and produced by Shanxi Zhongtong High Technology Co., Ltd. (Jinzhong, China) and Taiyuan University of Technology contains more than 30 wt%, and the thermal neutron-absorption properties are greater than 95%. The tensile strength of sheets is higher than 260 MPa, and it has a density of above 99.9%. The B_4_C/Al-based neutron-absorption plates with 5 wt% to 35 wt% B_4_C content are produced by Anhui Yingliu Jiuyuan Nuclear Energy New Material Technology Co., Ltd. (Hefei, China) in combination with Shanghai Institute of Nuclear Engineering Research and Design. With a maximum length of 4800 mm, a maximum width of 520 mm, and a thickness of 0.5~15 mm, the plate can be rolled, bent, and joint-welded, which can meet the service life of 60 years.

#### 3.3.1. Stirring Casting

**Figure 10:**
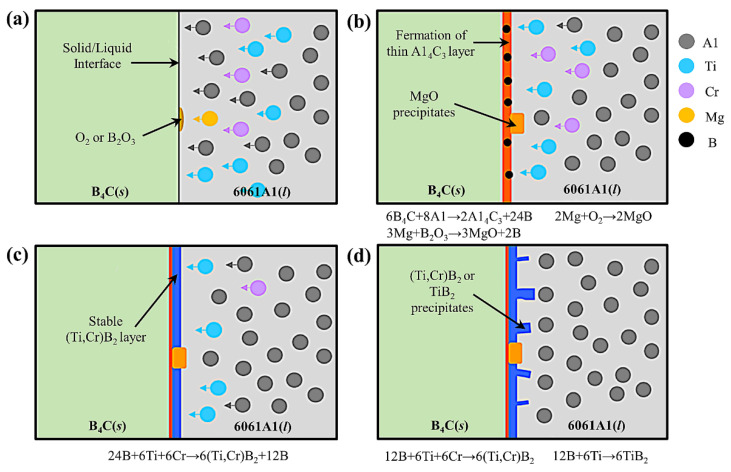
(**a**) Al atoms rapidly diffused toward B_4_C, (**b**) Al_3_BC phase generated at the B_4_C/Al interface, (**c**) (Ti,Cr)B_2_ layer was generated on Al_4_C_3_ layer and (**d**) TiB_2_ or (Ti,Cr)B_2_ precipitates were generated on (Ti,Cr)B_2_ layer [52].

The stirring casting process is simple and convenient to operate, and it is easy to control the composition [47]. The diameter size of B_4_C particles in stirring casting is in the range of 20~60 um; in this size range, B_4_C particles have remarkable granule state, are well dispersed and do not stick to the crucible [48], and they do not easily agglomerate towards the bottom of the crucible [49]. After entering the high-temperature aluminum melt, the B_4_C particles easily react with the aluminum matrix or are oxidized to B_2_O_3_ [50], resulting in poor interfacial adhesion and low wettability. Generally, Ti is added to improve the interfacial properties. The continuous multi-interface layer of (Ti, Cr)B_2_ and TiB_2_ formed by the B_4_C/Al interface can improve the wettability and bonding strength of B_4_C in molten Al. The relative density of 30 vol%/6061A1 can reach 100% and the tensile strength can reach 224.5 MPa after hot rolling [51]. Figure 10 shows a schematic diagram of the interfacial reaction between B_4_C/Al6061 alloy in stirring casting; the reaction between B_4_C and Al6061 can be considered as an atomic diffusion process. During stirring casting, Al atoms quickly diffuse into B_4_C and react to generate Al_4_C_3_ (Figure 10b). Then, the Ti and Cr atoms in the aluminum alloy diffuse into B_4_C and react with the remaining boron atoms in the interface layer to form a thermodynamically stable and continuous (Ti,Cr)B_2_ layer on the Al_4_C_3_ layer (Figure 10c). Finally, the remaining B and Ti (Cr) atoms react with Al atoms to form TiB_2_ or (Ti,Cr)B_2_ precipitates on the (Ti,Cr)B_2_ layer (Figure 10d). A small amount of unstable Mg atoms also diffuse to oxygen, and magnesia precipitates are formed on the Al_4_C_3_ layer of the B_4_C/Al interface [52].

In addition, compared with the traditional mechanical stirring, the magnetically coupled stirring and ultrasonic assisted stirring of B_4_C have higher turbulent kinetic energy than traditional mechanical stirring, the stirring dead zone is significantly reduced, and the B_4_C particles are more uniformly dispersed [53,54]. The content of B_4_C in casting is generally not more than 20 vol% so as not to affect the mechanical properties of the composites [51]. Moreover, the traditional stirring casting is prone to generate vertical eddy currents, which requires a special design of the crucibles or stirring rods, and the stirring environment is generally inert atmosphere or vacuum, which require high equipment.

#### 3.3.2. Powder Metallurgy

Powder metallurgy is the most commonly used method for preparing particle-reinforced metal matrix shielding materials, and the basic processes are powder preparation, mixing, molding, sintering, and deformation [55]. The utilization rate of raw materials is high, not limited by particle shape and type, and the structure and composition design is flexible [56].

**Figure 11:**
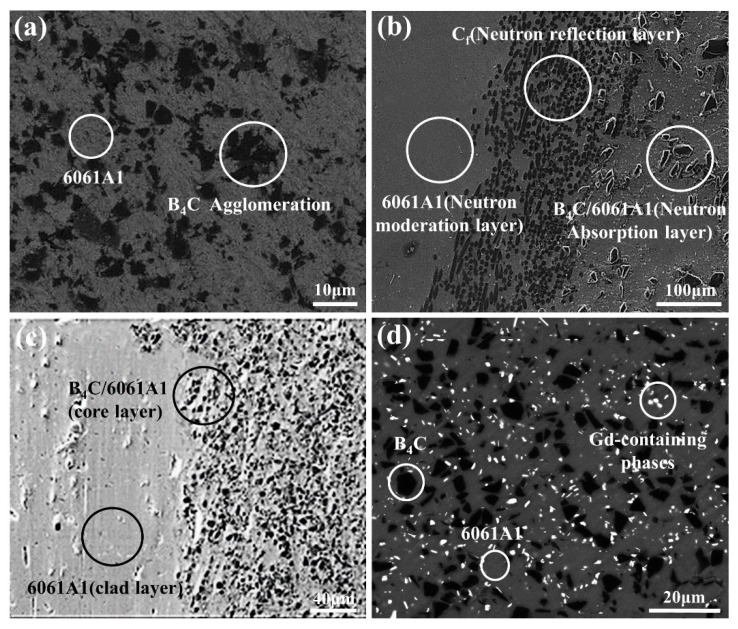
Microstructure of (**a**) 30 wt% B_4_C/6061A1 [57], (**b**) (B_4_C + 6061Al)/C_f_/6061Al [16], (**c**) 6061Al/(B_4_C + 6061Al)/6061Al [58], and (**d**) (1 wt% Gd + 15 wt% B_4_C)/6061Al [59].

Increasing the content of B_4_C particles can improve the neutron-absorption capacity of the composites. However, the addition of high content of B_4_C particles to the aluminum matrix will also cause a variety of problems, such as poor wettability between B_4_C and A1 matrix, bad interfacial reaction, and agglomeration (Figure 11a) [57]. Therefore, it is difficult to prepare neutron-absorbing materials with high B_4_C content and without the above defects. The content of B_4_C particles can be increased by novel structural design and manufacturing process, such as (20 wt% B_4_C + 6061A1)/C_f_/6061A1 layered neutron-absorption material prepared by spark plasma sintering. The moderation layer, absorption layer, and reflection layer are closely connected (Figure 11b). The neutron escaped from the absorption layer can be reflected back into the absorption layer for repeated absorption by C_f_, and the neutron-absorption rate is up to 86%. The high modulus and axial strength of C_f_ make the tensile strength and strain of the composites reach 245 MPa and 14% [16]. The sandwich 6061Al/(30 wt% B_4_C + 6061Al)/6061Al composite (Figure 11c) prepared by hot isostatic pressing has good wettability with 6061Al, strong interfacial adhesion, low porosity, and significantly eliminates internal defects, and the neutron absorptivity is 98.6% [58].

Higher B_4_C content is beneficial to the higher neutron-shielding property and strength of the composites, but not conducive to the plasticity and toughness of the composites. If the elements with higher neutron-shielding properties are selected to replace the elements with lower neutron-shielding properties, the neutron-shielding properties of the materials can be ensured and the plasticity and toughness of the materials can be improved while reducing the content of neutron-absorbing elements. Some studies have shown that the neutron-absorption property of (15 wt% B_4_C + 1 wt% Gd)/Al material prepared by replacing part of B_4_C with Gd with the largest neutron-absorption cross section (Figure 11d) is similar to that of 30 wt% B_4_C/A1, the thermal neutron-absorption rate is more than 99%, the mechanical properties are better than 30 wt% B_4_C/A1, the tensile strength is 400 MPa, and the elongation reaches about 5% [59]. However, Gd will produce very high energy gamma rays (about 8 MeV) while absorbing neutrons, and additional shielding of gamma rays is needed [60]. W has neutron- and gamma-ray-shielding properties, especially excellent shielding properties for gamma rays. The new (Gd_2_O_3_ + W)/A1 composites developed by combining Gd and W have excellent neutron- and gamma-ray- double-shielding properties [15]. In order to make the composites have good plasticity and toughness, the content of B_4_C reinforced particles in the powder metallurgy process is generally less than 30 wt%.

According to research findings, the interfacial reaction between Al and B_4_C in powder metallurgy is a factor that must be considered. The Al–B_4_C system produces different phases under temperatures and times, which exert different effects on the material properties. Table 4 presents the reaction products and physical properties of the Al–B_4_C system [61], in which AlB_2_ exists in the form of rod-like crystals, which can resist the external force deformation and improve mechanical properties [62]; AlB_2_ is also in a phase with high hardness and excellent wear resistance, which can improve the micro hardness of composites [63].The Al_3_BC nanoparticles can improve the grain boundary bonding strength, and the interaction between single Al_3_BC nanoparticles and dislocations will lead to a significant improvement in composite strength [64].

The powder metallurgy is unable to prepare large-scale products due to the limited size of the sintering furnace; only a slight increase in the longitudinal and transverse lengths can be obtained by subsequent plastic deformation, and the industrial applications is poor. B_4_C is mainly bonded and has a low atomic self-diffusion rate, which makes it difficult to sinter densification extremely [65], resulting in low strength, plasticity of the composites, and high internal porosity. It is necessary to reprocess the prepared product. With the development of sintering technology, powder metallurgy has more unique advantages in the field of nuclear-shielding materials.

#### 3.3.3. Infiltration Process

**Figure 12:**
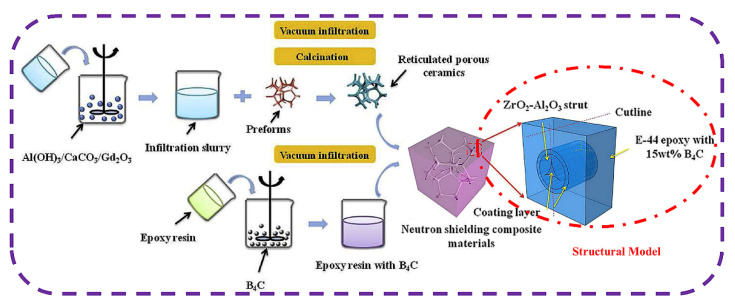
Schematic diagram of fabrication of three-dimensional interlaced neutron-shielding materials [68].

The infiltration method is to prepare the powder particles into the required ceramic skeleton structure in advance, which form a connected channel inside, then under the conditions of pressure or no pressure, the molten metal is spontaneously infiltrated into ceramic skeleton through capillary force to form a composite material. The infiltration process has been widely used in the preparation of complex and customized nuclear shielding materials, e.g., the neutron collimator of B_4_C material is manufactured by 3D printing, the collimator is infiltrated by Al, and a collimator of B_4_C/Al material with a consistency of 97% can be prepared, which is applied without reprocessing, breaking the restriction that neutron-absorption materials can only be made of plates [66]. The porous B_4_C–TiB_2_ ceramic skeleton is prepared by directional freezing, allowing the B_4_C/Al shell-like layered composite material to be obtained by infiltrating and melting A1 into the ceramic skeleton at low temperature and low pressure. TiB_2_ has a strong bond with A1 and B_4_C. The specific strength and toughness of the material have reached the level of titanium alloys, which has potential application in the field of nuclear industry [67]. In addition, the epoxy-resin-modified B_4_C is vacuum infiltrated into the ceramic framework containing Gd_2_O_3_ to form a three-dimensional interwoven ceramic/epoxy composite containing double neutron-absorbing nuclides (Figure 12), which exhibit maximum compressive strength of 71.14 MPa and thermal conductivity of 2.32 W/(m·K) with fast neutrons and thermal neutron-shielding of 26.3% and 89.3%, respectively. It is expected to be applied in nuclear reactors [68].

The advantages of B_4_C/Al composites prepared by infiltration process are weak adverse interfacial reactions and near-net molding products, which manufacture high content of B_4_C and large size, complex parts, and the process is simple and inexpensive [69]. The composites prepared by this process have high density and are continuous in microstructure with desirable comprehensive mechanical properties. However, the wettability between B_4_C and Al matrix is poor below 1200 °C. Generally, it is necessary to increase the immersion temperature by more than 1200 °C, or even higher, but the increase in immersion temperature will cause the formation of an impurity phase in the interface reaction between them and lead to impurity phase and easy oxidation of B_4_C, which will reduce the properties of the composites [70]. There is no doubt that finding a way to prepare B_4_C framework with uniform pore distribution is a difficulty faced by researchers.

#### 3.3.4. New Materials

The preparation methods of composite B_4_C/Al are not limited to the above methods, but also include mechanical alloying, spray deposition, self-spreading high-temperature synthesis, etc., or a combination of several fabrication methods. The structure of the material also evolves from homogenous multi-dimensional to spatially multi-dimensional, and these particular fabrication processes combined with construction make the composites exhibit more favorable comprehensive properties.

**Figure 13:**
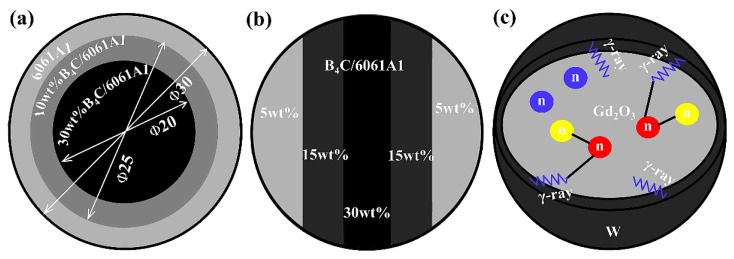
Schematic diagram of (**a**) (B_4_C + 6061Al)/6061Al concentric ring structure [71], (**b**) B_4_C/6061Al laminar composites [72] and (**c**) Gd_2_O_3_@W/A1 core–shell structure [73].

In addition to the previous introduction of B_4_C/Al-based composites, B_4_C/Al-based new materials include the following: Figure 13a shows a B_4_C/6061Al neutron-absorption material with “annual ring” structure with different content and layer thickness. After hot pressing and sintering, the subsequent extrusion and rolling deformation can be carried out. After extrusion and rolling deformation, the distribution uniformity and mechanical properties of B_4_C particles are improved, and the content of B_4_C particles increases gradually from the outside to the inside. The structure can also be applied to the preparation of composites with external strength and internal toughness, such as the preparation of wear-resistant materials, impact-proof materials, etc., and the particle content decreases gradually from the outside to the inside [71]. Figure 13b is a lamellar B_4_C/Al6061 neutron-absorption material with different B_4_C particle content. The structure can obtain excellent mechanical properties after extrusion and rolling, and the tensile strength can reach 260 MPa with good plastic formability [72]. Figure 13c shows Gd_2_O_3_@W/Al neutron- and gamma-ray-double-shielding materials with core–shell structure. Gd_2_O_3_@W core–shell particles are prepared by coating tungsten on the surface of Gd_2_O_3_ particles. The tungsten shell can shield not only the primary gamma rays released from the external spent fuel, but also the secondary gamma rays excited by the internal Gd atoms when absorbing neutrons, thus realizing the double-shielding function of primary gamma rays and secondary gamma rays. In addition, the core–shell can also prevent crack propagation and further improve the mechanical properties of the material [73]. These new structural materials not only maintain good neutron-absorption properties, but also significantly improve the specific properties of the composites and have a good potential application prospect.

**Figure 14:**
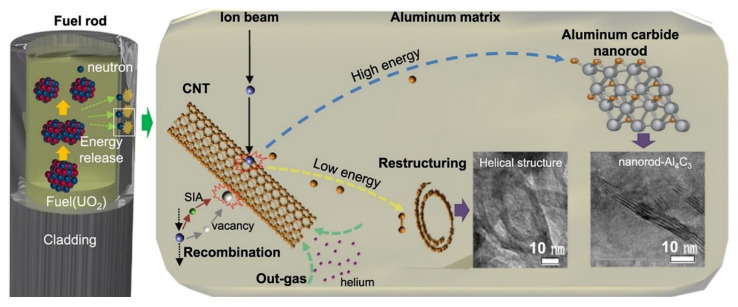
Schematic diagram of irradiation process for CNT/A1 materials [17].

Shielding materials exposed to high-energy neutrons and gamma rays will lead to the formation of radiation defects, such as dislocation rings, stacking fault tetrahedrons, bubbles, amorphous substances, or radiation-induced segregation. These irradiation defects are usually accompanied by a decrease of the physical and mechanical properties of materials and early failure of components, which are also the key factors to accelerate the failure of components [74]. For example, radiation causes metal atoms to shift from their equilibrium lattice positions, resulting in lattice defects, which in turn leads to an increase in hardness, but also embrittlement, thus reducing ductility [75]. The study on the radiation resistance of carbon nanotubes (CNT) in aluminum shows (Figure 14) that dispersed CNT can improve the tensile strength of metallic materials without reducing ductility, and metastable Al_4_C_3_ can reorganize radiation defects, self-heal, and reduce void/pore generation and radiation embrittlement. In addition, carbon nanotubes with high aspect ratio provide a rich interface in contact with the matrix, and the hollow structure is conducive to the release of fission gas [17]. The shielding material with carbon-like nanotube self-repairing radiation damage has great potential.

### 3.4. Polymer-Based Materials

Currently, polymer matrix composite shielding material is one of the most common shielding materials. The matrix polymer contains a large number of hydrogen elements, which effectively attenuate high-energy neutrons and weaken gamma radiation, compared with tradition metals through elastic scattering. Since there are no neutrons in the hydrogen nucleus, hydrogen also offers the additional benefit of no secondary neutron radiation. Polymer-based composites matrix has great application potential in neutron-shielding material because it is lighter, more flexible, and lower in cost than the most promising neutron-shielding materials.

Polyethylene (PE) is the most commonly used thermoplastic matrix polymer for neutron-shielding materials. As shown in Table 5, high-density polyethylene (HDPE) is compared with low-density polyethylene (LDPE). HDPE has higher tensile strength and melting point, especially hydrogen content as high as 14.4 wt%, showing excellent fast neutron-moderation properties, so it is considered to be an ideal polymer-based radiation-shielding substrate. LDPE has the advantage of lower coefficient of thermal expansion; the use of LDPE with embedded B_4_C particles is an alternative to obtain a flexible and lightweight material. B_4_C, BN, Gd_2_O_3_, WO_3_, and Sm_2_O_3_ with larger neutron-absorption cross section are usually used as reinforced particles of PE [76,77,78], but the small contact angle between these particles and PE matrix leads to poor adhesion and compatibility between these particles and matrix, which is easy to agglomerate, so it is usually required to modify the surface with alkoxysilane [79]. The composites showed excellent radiation shielding, thermal conductivity, and mechanical properties [80]. It has also been studied to improve the mechanical properties of PE-based composites by adding nanometer or submicron reinforced particles, e.g., even the very low amount (0.6~1.7 wt%) of incorporated nano/submicron B_4_C particles in LDPE matrix improved the neutron shielding (up to 39%), tensile strength (9.3%), and impact resistance (8%) of the composites [81]. In addition, the properties of the composites can also be improved by innovating the material structure, such as the sandwich structure neutron-absorption composite developed by carbon fiber and boron carbide [82] and the HDPE/h-BN/LDPE multilayer alternating structure neutron-absorption materials developed by h-BN, which only have good neutron-absorption properties, but the mechanical properties and thermal stability have also been significantly improved [83]. However, the structural strength and thermal stability of polyethylene-based shielding materials are poor when the service temperature is higher than 100 °C, which limits its application at high temperature [84].

Epoxy (EP) is a polymer of a thermosetting matrix with friendly adhesions to the reinforcing particles and good mechanical properties after curing. It especially has good elastic scattering and stability for reactor neutrons with degassing characteristics, and good resistance to gamma-ray radiation with excellent durability [86]. EP is always used in combination with other reinforcements (such as fibers, whiskers, and particles). The average size, aspect ratio, and content of reinforcements are the key factors affecting the properties of EP matrix composites. A small amount of reinforcements can improve the mechanical properties of the composites [87]. At higher contents, deposition and agglomeration easily occur, and ultrasonic dispersion treatment is generally used to improve the uniformity of the reinforcement in the matrix, so that they have good wettability and effective improvement of the interfacial adhesion, thus improving the mechanical and neutron-absorption properties of the material [88]. The addition of B_4_C to EP resin affects the curing process to a lesser extent, but it affects (accelerating or retarding) the kinetic reactions, reducing the glass transition temperature, depending on the B_4_C particle size and amount [89]. Metal–organic frameworks (MOFs) with shielding properties such as Gd and B play a key role in toughening EP matrix composites, too. MOF/EP materials have better thermal and mechanical properties than pure EP, which provides an idea for improving the properties of radiation protection materials [90,91].

**Figure 15:**
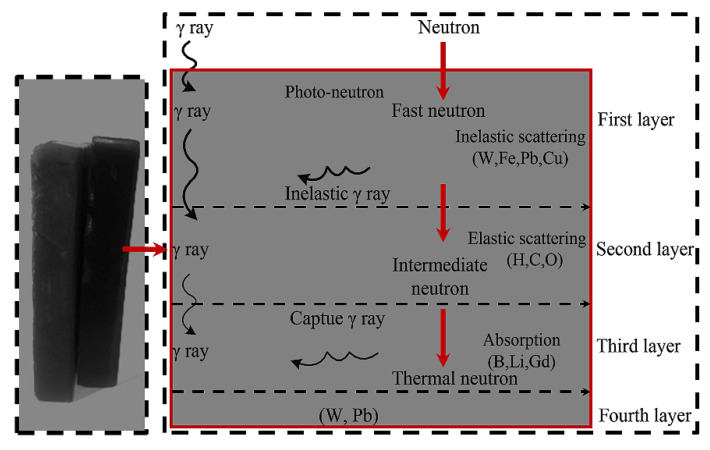
Interaction of neutrons and gamma rays in the multi-layer material [94].

The study proved that the neutron-shielding effect of multilayer gradient materials with different reinforcements based on EP is obviously better than that of uniform mixed materials. As shown in Figure 15, the first layer selected elements with high inelastic scattering cross section to slow down fast neutrons. In the second layer, the elements with high elastic cross section values are selected to convert moderated neutrons into low-energy thermal neutrons. The elements with high thermal neutron-absorption cross section are selected in the third layer, and the elements with high-gamma-ray-absorption cross section are selected in the fourth layer, to shield the primary gamma rays and secondary gamma rays. This kind of gradient material has the advantages of low density, light weight, compact structure, and strong pertinence, which can be applied to shield material for the transportation of spent fuel [92].

Polyimide resin (PI) has a very stable aromatic heterocyclic structure, which made it exhibit incomparable thermal (decomposition temperature 600 °C, long-term use at 330 °C) and low-temperature resistance that other polymers could not achieve (−269 °C without embrittlement). The mechanical properties are excellent and the tensile strength can reach 400 MPa. PI has high radiation resistance, and the tensile strength retention rate of the film after 5 × 10^7^ Gy irradiation is 90% [19]. Table 6 shows the performance comparison of common reinforcements PI-based neutron-shielding materials. It can be seen that PI-based-film shielding materials have outstanding properties and have great potential application value in the field of nuclear shielding flexibility. Moreover, the wettability between polyimide resin and carbon fiber is very good, and the reinforcement contains neutron-absorption elements, high specific strength, high specific modulus, and excellent high-temperature properties.

Ethylene propylene diene monomer (EPDM) is a saturated polymer chain with polyethylene, including ethylene, propylene, and diene monomers, that is mainly used in the industry as commercial rubber, and the price is cheap. Due to the obvious effect of high hydrogen content on neutron aging, EPDM usually acts as a matrix material to mix with neutron absorbers and plays a role in neutron aging and absorption [12]. The addition of B_4_C can form thermally conductive channels with high thermal conductivity, which can help to reduce the heat accumulation generated by cyclic deformation and extend the fatigue life and service time of rubbers [94]. The addition of tungsten (W) significantly enhances neutron- and gamma-ray-shielding properties [95]. The addition of h-BN can increase the elastic modulus, decrease the elongation at break, and increase the glass transition temperature. The composites have good thermal properties and thermal neutron-shielding properties [11]. The MCNPX simulation showed that the thermal neutron-absorption coefficient of the composites doubles with the increase of 5 wt% B_4_C [96]. However, EPDM- and EP-based materials cannot withstand temperature above 170 °C [59], especially in the event of radiation accidents, and they may lose their protective effect.

The reinforcements in polymer-based composites have poor adhesion and weak adhesion to the matrix due to their strong surface hydrophobicity, which can easily lead to the deterioration of the mechanical properties of polymer-based materials, which usually need to be treated as follows: (1) surface modification and directional function of reinforced particles, (2) making the reinforced particles nanometer-sized, (3) added materials with high aspect ratio or three-dimensional structure, such as carbon nanotubes, carbon fibers, and so on. In addition, polymer-based composites have unstable molecular chain size, and easily undergo aging and radiation damage under high-temperature and acid–alkaline environments; thus, there are fire hazards with flammability.

### 3.5. Shielding Concrete

Shielding concrete derives its radiation protection properties from modification by common concrete components, which are highly flexible in compositional design and inexpensive to build and maintain. The radiation attenuation in concrete mainly depends on the type of aggregate, primarily water–cement ratio, element composition, moisture content, and density [97].

Traditional high-density iron aggregates (hematite, limonite, etc.) and lead aggregates (galena, etc.) can easily lead to concrete segregation, high heat of hydration, and significant activation. Usually, small density, fine particle size, and elements with high neutron-absorption cross section are added as admixtures and aggregates, such as nanometer-scale B_4_C, Gd_2_O_3_, PbO_2_, FeB, Fe_2_B, WO_3_, and other reinforced particles [18,98,99,100], which can significantly improve the gamma- and neutron-radiation-shielding ability of concrete and reduce the weight of concrete [101]. However, such substances have poor compatibility and segregation in the concrete matrix, which leads to decreased flow plasticity and slump, reduced compressive and tensile strength, easy cracking, and poor durability. Generally, the content should not undergo too much modification, and surface treatments are required.

**Figure 16:**
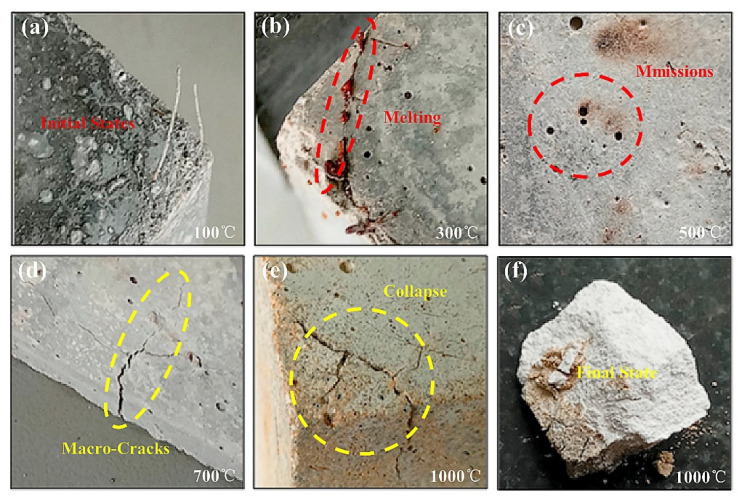
Details of the macrostructure after exposure to high temperatures at (**a**) 100 °C, (**b**) 300 °C, (**c**) 500 °C, (**d**) 700 °C, (**e**,**f**) 1000 °C [102].

The incorporation of polymer fibers with abundant hydrogen content in concrete can effectively improve the mechanical properties and shielding efficiency of concrete and also reduce the weight of concrete. For example, the most common added fibers are polyethylene ((C_2_H_4_)_n_), polypropylene ((C_3_H_6_)_n_), or polyvinyl alcohol ((C_2_H_4_O)_n_), which can provide more H content, the neutron moderation effect is significant, and the gamma-ray-shielding rate can be doubled. (C_2_H_4_O)_n_ has higher density and melting point (200 °C), so its use is more appropriate when high temperatures are generated by spent fuel. Furthermore, temperature also plays a crucial role in determining its shielding properties. As shown in Figure 15, polymer-modified concrete melted and volatilized at 300~500 °C, cracked at 700 °C, collapsed at 1000 °C, and the compressive strength was reduced by 90% at 500~1000 °C (Figure 16) [102]; therefore, it requires cumbersome mechanical cooling systems, which limit its application in high-temperature environments.

## 4. Conclusions

In the transportation and storage of spent fuel, neutron-shielding materials are mainly categorized into metal-based composites, polymer-based composites, and shielding concretes. Metal-based neutron-shielding materials are applied in the regions where high strength, temperature, and durability are required. Polymer-based neutron-shielding composites have the advantages of light weight, easy manufacture, uniform distribution of neutron absorbers, and good chemical corrosion resistance, which are mainly used to shield curved and irregular surfaces. Shielding concretes show enhanced attenuation toward very fast neutrons.

Dual-use shielded containers for spent fuel transportation and storage is the main direction of future development of spent fuel storage, and dry storage of spent fuel is also necessarily a development trend. The best material not only depends on its nuclear-radiation-shielding properties, but also takes into account its specific use environment and economic and other requirements. The future development trend of spent fuel transportation and storage neutron-shielding materials should have the following characteristics:

- Rare-earth monomers and their oxides have large neutron-absorption cross section values, which can effectively avoid the formation of He bubbles in the radiation capture reaction, prolong the service life of neutron-absorbing materials, and increase the storage capacity of spent fuel. There is a great potential for the development of new-structure, functionally integrated, neutron-absorbing materials using rare-earth monomers or their oxides as fillers, and two or more fillers acting together in combination to obtain new properties.
- The radiation shielding performance is strong. It not only has the functions of slowing down and absorbing neutrons, but also has high shielding performance for the primary gamma rays released by spent fuel and the secondary gamma rays released by the interaction between target nucleus and neutron in shielding materials. It can shield neutrons and gamma rays at the same time. In the process of use, the three-dimensional size of the shielding material is stable and has good compatibility with other related material components. It is easy to replace and repair when it is necessary to replace or repair.
- Excellent mechanical properties, sufficient structural performance, and strength for extremely harsh environments. The utility model has the advantages of high thermal stability, corrosion resistance, light weight, simple production process, low cost, easy processing, environmentally friendly, and long service cycle. The release rate of fissile gas generated by spent fuel is high, it has strong resistance to radiation damage, and it has a certain self-healing function for irradiation damage.

## Figures and Tables

**Table 1:** Cross section values of a common neutron absorber in the spent fuel field [7].

Z	Symbol	Name	A (u)	σ_cs_ (barn)	σ_is_ (barn)	σ_s_ (barn)	σ_a_ (barn)
64	Gd	Gadolinium	157.3	29.388	151.222	180.22	49,700
62	Sm	Samarium	150.4	0.422	39.333	39.33	5922
63	Eu	Europium	152.0	6.754	2.544	9.24	4530
48	Cd	Cadmium	112.4	3.046	3.461	6.50	2520
66	Dy	Dysprosium	162.5	35.988	54.412	90.39	994
5	B	Boron	10.80	3.545	1.701	5.24	767
74	W	Tungsten	183.85	2.97	1.63	4.6	18.3
28	Ni	Nickel	58.69	13.3	5.2	18.50	4.49
24	Cr	Chromium	51.996	1.66	1.83	3.49	3.05
26	Fe	Iron	55.8	11.225	0.401	11.62	2.5633
13	Al	Aluminum	27.0	1.495	0.008	1.50	0.2313

σ_a_: absorption cross section for 2200 m/s neutrons (E ¼ 25.3 MeV).

**Table 2:** Performance comparison of several main neutron-absorption fillers.

Compound	Neutron Absorption	Behavior under High Temperatures	Chemical Resistance	Mechanical Strength	Usage	Ref.
B_4_C	High	Excellent	Excellent	Excellent	Low density; very expensive; can be added to any matrix; low elastic modulus and tensile strength	[10]
h-BN	Medium–high	Excellent	Excellent	Poor	Low density; very expensive; an ideal additive for high energy neutron decay polyethylene and polyimide	[11]
H_3_BO_3_	Medium	Very poor	Good	Poor	Lowest density; cheap; usually used in combination with polymer matrix composites	[12]
Gd/Gd_2_O_3_	Highest	Excellent	Very good	Good	High density; commonly used in metal-based shielding materials	[13]
Sm_2_O_3_	Very high	Excellent	Very good	Good	High density; medium; commonly used in polymer groups	[14]
W/WO_3_	Very low	Excellent	Very good	Good	Very high density; expensive; auxiliary absorption of gamma rays	[15]
Carbon Fiber	Very low	Excellent	Excellent	Excellent	Very low density; very expensive; excellent nuclear reflective material	[16]
Carbon Nanotubes	Very low	Excellent	Excellent	Excellent	Very low density; very expensive; commonly used in metal-based shielding materials	[17]
Fe–B	Medium	Medium	Good	Good	Added to concrete as a promising shield material for the fast neutron application	[18]

**Table 3:** Standard composition and mechanical properties of the B/A1 alloy [42].

Element Content (wt%)	B	Cu	Si	Fe	Mg	Mn	Zn	Ti	Others
AA1100	1.25–4.5	0.05–0.20	1.0 (Si + Fe)		-	0.05	0.01	-	0.10 total
AA6351	2.0	0.10	0.7–1.3	0.50	0.4–0.8	0.4–0.8	0.02	2.4 × B	0.15 total
	Temperature (°C)		Elongation (%)		Yield Strength (MPa)		Tensile Strength (MPa)		
	25		10		276		310		
	200		12		193		214		

**Table 4:** Physical properties of each reaction product of the Al–B_4_C system [61].

Phase	Microhardness (kg/mm^3^)	Density (g/mm^3^)	Formation Temperature (°C)
A1	19	2.70	-
B_4_C	2750~4950	2.52	-
Al_3_BC	1400	-	450
AlB_2_	980	3.16	600
AlB_24_C_4_	2530~2650	2.54	1000
Al_4_C_3_	1250	2.93	1000+

**Table 5:** HDPE and LDPE performance comparison [85].

Material	Attenuation Coefficient Σ (cm^−1^)	Density (g/cm^3^)	Coefficient of Thermal Expansion (10^−5^/°C)	Melting Point (°C)	Tensile Yield Strength (MPa)	Tg (°C)
HDPE	0.145	0.96	15.3	130	26.3	−110
LDPE	0.330	0.91	2.0	114	11.5	−110

**Table 6:** Performance comparison of PI-based neutron-shielding materials.

Shielding Materials	Decomposition Temperature (°C)	Tensile Strength (MPa)	Neutron Permeability/% (Thickness/cm)	Ref.
C_f_ reinforced 21 wt% Sm_2_O_3_/PI	300	200	50% (1 cm)	[78]
(3 wt% h-BN + 3 wt% Gd_2_O_3_)/PI	-	78	70% (1 cm)	[74]
3 wt% Gd–MOF/PI	568	75	14% (1 cm)	[93]
30 wt% B_4_C/PI (films)	622	406	24% (0.08 cm)	[5]

## Data Availability

Not applicable.
